# Draft genome sequencing of multidrug-resistant *Pseudomonas putida* strains, isolated from dairy cows with clinical mastitis and their farm environment

**DOI:** 10.1128/mra.00886-24

**Published:** 2024-10-15

**Authors:** Tima Tisa Mallick, Naim Siddique, Md. Morshedur Rahman, Khaled Hassan Shuvo, Ziban Chandra Das, M. Nazmul Hoque

**Affiliations:** 1 Molecular Biology and Bioinformatics Laboratory, Department of Gynecology, Obstetrics and Reproductive Health, Bangabandhu Sheikh Mujibur Rahman Agricultural University (BSMRAU), Gazipur, Bangladesh; The University of Arizona, Tucson, Arizona, USA

**Keywords:** draft genomes, *Pseudomonas putida*, multidrug resistance, virulence factors, mastitis, dairy cows

## Abstract

We sequenced the genomes of three multidrug-resistant *Pseudomonas putida* strains namely 11CM-M1, 11CM-F1, and 11CM-S1, isolated from milk, feces, and farm soil samples collected from dairy cows with clinical mastitis. The assembled draft genomes of these *P. putida* strains were approximately 5.6 Mbp, with a GC content of 62.4%.

## ANNOUNCEMENT

Mastitis, an inflammation of the mammary gland, is one of the most prevalent diseases impacting dairy cows globally (1). The disease is caused by a plethora of microorganisms, including major pathogens (2, 3), as well as emerging and opportunistic bacteria (4, 5). *Pseudomonas* spp. are opportunistic pathogens that can cause severe mastitis in dairy animals, frequently associated with environmental contamination and inadequate hygiene practices (6). *Pseudomonas putida*, a widely dispersed evolutionary group within the genus *Pseudomonas*, evades and exploits host immunity to colonize mammary tissues, leading to clinical mastitis (7).

Milk, feces, and farm soil samples from a cow diagnosed with clinical mastitis (CM) using the California Mastitis Test (CMT) (8) were collected from a dairy farm located in the Gazipur district (24.09° N, 90.41° E) of Bangladesh. A total of 110 samples were collected, including 45 from CM milk, 35 from feces, and 30 from farm soil, representing a variety of small- and large-scale dairy operations. Milk samples were obtained following the standards set by the National Mastitis Council in 2014 (9). Only freshly dropped, wet fecal materials were collected from the ground. Approximately 10 mL of milk and 5 g of feces and soil were collected from each cow in sterile Falcon tubes during morning milking (8:00–10:00 a.m.), with careful attention to pre-sampling disinfection of teat ends and strict sampling hygiene. For isolation and phenotypic identification of *P. putida*, samples were enriched in nutrient broth (Oxoid, UK) and incubated overnight aerobically at 37°C. Thereafter, the bacteria-enriched broth was serially diluted up to 10^−3^ fold, plated on nutrient agar (Oxoid, UK), and incubated overnight at 37°C. Pure colonies were obtained by streaking on selective tryptic soy agar (TSA) (Oxoid, UK) plates by incubating at 37°C for 24 h (10). The isolates were phenotypically identified by examining their colony morphology and assessing the results of Gram staining (Gram-negative, rod shape, pink colony) (11). VITEK-2 system v9.01 was used to perform the species-level identification of *P. putida* (12). The 11 CM-M1, 11 CM-F1, and 11 CM-S1 isolates of *P. putida* showed resistance to oxacillin, ampicillin, nalidixic acid, aztreonam, cefoxitin, and tetracycline, according to Kirby-Bauer disk diffusion tests following CLSI guidelines (13). A single pure colony from each multidrug-resistant *P. putida* isolate was inoculated into the nutrient broth and incubated overnight at 37°C. DNA was then extracted using the QIAamp DNA Mini Kit (QIAGEN, Germany) (14). Whole-genome sequencing libraries were constructed from 1 ng of DNA (from each isolate) using the Nextera DNA Flex Kit (Illumina, USA). The libraries were sequenced on an Illumina MiSeq platform, employing a 2 × 250 bp paired-end read configuration. Raw reads underwent trimming with Trimmomatic v0.39 (15) and quality checking through FastQC v0.11.7 (16). The draft genomes were assembled using SPAdes v3.15.5 (17) and annotated using the NCBI Prokaryotic Genome Annotation Pipeline v6.6 (18). Genome completeness, virulence factor genes (VFGs), antimicrobial resistance genes (ARGs), and metabolic functions in the draft genomes were predicted using CheckM v1.2.2 (19), VFDB v6.0 (20), ResFinder 4.0 (21), and RAST server (22), respectively. In all analyses, default parameters were used unless otherwise specified.

**TABLE 1:** General genomic features of the *P. putida* strains isolated from milk, feces, and farm soil of clinical mastitis cows

Genomic features	*P. putida* strains		
	11 CM-M1	11 CM-F1	11 CM-S1
No. of reads	1,380,818	1,180,802	1,266,200
Genome size (bp)	5,615,898	5,614,984	5,616,715
Genome coverage (×)	52	45	50
Genome completeness (%)	99.35 (0.19% contamination)	99.35 (0.19% contamination)	99.35 (0.19% contamination)
No. of contigs	40	42	43
Contig *N*_50_ (bp)	328,801	269,713	269,713
GC content (%)	62.4	62.4	62.4
Coding sequences (CDSs)	5,122	5,119	5,119
Protein-coding genes	5,075	5,072	5,072
No. of SEED subsystems	367	367	367
Genome accessions	JAXGFH000000000	JAXGFF000000000	JAXGFG000000000
SRA accessions	SRR26980303	SRR26980301	SRR26980302

The draft genomes of the multidrug-resistant *P. putida* strains 11 CM-M1, 11 CM-F1, and 11 CM-S1 were approximately 5.6 Mbp in size with a GC content of 62.4% and 99.35% genome completeness. They were assembled into 40, 42, and 43 contigs, respectively, with genome coverages of 52×, 45×, and 50×. Each genome exhibited 367 SEED subsystems related to metabolic features and included 78 RNA genes. In addition, they were found to harbor 36 VFGs and nine ARGs, indicating resistance to multiple antibiotics. The genomes of multidrug-resistant *P. putida* strains from mastitis-affected dairy cows reveal significant antibiotic resistance and virulence potential, emphasizing the need for targeted interventions for mastitis in dairy farming.

## Data Availability

The whole-genome shotgun project of the *P. putida* strains has been deposited in GenBank and the NCBI Sequence Read Archive (SRA) under BioProject accession number PRJNA1044409. The SRA accessions are listed in Table 1. The versions described in this paper are version JAXGFH000000000.1, JAXGFF000000000.1, and JAXGFG000000000.1.
